# Personalized Circulating Tumor DNA Assay to Assess Long-Term Clinical Benefit in Patients with Advanced Melanoma

**DOI:** 10.3390/cancers17233804

**Published:** 2025-11-27

**Authors:** Clara Martínez-Vila, Cristina Teixido, Roberto Martín, Francisco Aya, Sumedha Sudhaman, Griffin L. Budde, Europa Azucena González-Navarro, Llucia Alos, Natalia Castrejon, J. Bryce Ortiz, Michael Krainock, Minetta C. Liu, Ana Arance

**Affiliations:** 1 Department of Medical Oncology, Hospital Clínic of Barcelona, Villarroel 170, 08036 Barcelona, Spain; cmartinezv@althaia.cat (C.M.-V.);; 2 Programa de Doctorat en Medicina i Recerca Translacional, Facultat de Medicina i Ciències de la Salut, Universitat de Barcelona (UB), c. Casanova, 143, 08036 Barcelona, Spain; 3 Molecular Biology CORE Laboratory, Biomedical Diagnostic Centre (CDB), Hospital Clínic, Villarroel 170, 08036 Barcelona, Spain; 4 Department of Pathology, Hospital Clínic of Barcelona, University of Barcelona, Villarroel 170, 08036 Barcelona, Spain; 5 Grupo Español de Terapias Inmuno-Biológicas en Cáncer (GÉTICA), Velázquez, 7-3º Planta, 28001 Madrid, Spain; 6 August Pi i Sunyer Biomedical Research Institute (IDIBAPS), Rosselló 149, 08036 Barcelona, Spain; 7 Natera, Inc., Austin, TX 78753, USA; 8 Department of Immunology, Hospital Clínic of Barcelona, University of Barcelona, Villarroel 170, 08036 Barcelona, Spain

**Keywords:** advanced melanoma, ctDNA, personalized assay, long-term outcomes, anti-PD-1, immunotherapy, biomarker, treatment duration

## Abstract

**Background**: Immune checkpoint inhibitors (ICIs) targeting PD-1 have significantly improved outcomes in patients with advanced melanoma. However, the optimal treatment duration remains undefined. Circulating tumor DNA (ctDNA) has emerged as a promising biomarker for treatment response monitoring and surveillance and can predict long-term clinical outcomes. **Methods**: Clinical and ctDNA data from prospectively enrolled patients with stage IV melanoma treated with anti–PD-1-based therapy at a single academic center were retrospectively analyzed. Of the 56 patients eligible, 28 underwent serial ctDNA testing during ICI treatment and follow-up (median 31 months) using a personalized, tumor-informed assay. Landmark analysis at 6 and 9 months was performed to assess progression-free survival (PFS) based on ctDNA status. Multivariable Cox regression was used to identify independent predictors of long-term outcomes. **Results**: Pre-ICI treatment, 91.7% (11/12) of evaluable patients were ctDNA-positive. At 6 months, ctDNA negativity or clearance was observed in 47.4% (9/19), and was strongly associated with improved PFS in the landmark analysis (HR: 10.0, *p* = 0.03; 2-year PFS: 89% in ctDNA-negative versus 30% in ctDNA-positive groups). At 9 months, persistent ctDNA positivity trended toward worse PFS. Multivariate analysis confirmed ctDNA status at the 6-month landmark timepoint to be an independent predictor of long-term benefit. **Conclusions**: Tumor-informed ctDNA testing is a robust, non-invasive tool to predict long-term benefit from anti–PD-1-based therapy in advanced melanoma. ctDNA clearance or sustained negativity at 6 months may serve as a surrogate for durable response and could inform individualized treatment discontinuation strategies, and minimize toxicity and cost while maintaining efficacy. These findings, derived from a limited single-center cohort, warrant further exploration and validation in larger studies.

## Simple Summary

Immune checkpoint inhibitors have transformed the management of advanced melanoma, increasing survival for many patients. However, the optimal duration of therapy remains unclear, as prolonged treatment may increase toxicity and cost without additional benefit. In this study, we evaluated a personalized, tumor-informed circulating tumor DNA (ctDNA) assay for monitoring treatment response and predicting long-term benefit. Among patients receiving immune checkpoint inhibitors, the absence or clearance of ctDNA six months after treatment initiation was strongly associated with improved survival. These results suggest that molecular monitoring through personalized, tumor-informed ctDNA analysis may identify patients who have achieved a durable response. Incorporating this approach into clinical practice could help guide individualized decisions on treatment duration, enabling therapy discontinuation in selected patients while maintaining efficacy and minimizing unnecessary exposure.

## 1. Introduction

Immune checkpoint inhibitors (ICIs) have transformed the treatment landscape for advanced melanoma. Anti-programmed cell death-1 (anti-PD-1) monoclonal antibodies such as nivolumab and pembrolizumab, either as monotherapy or in combination with agents targeting cytotoxic T-lymphocyte-associated protein 4 (anti-CTLA-4) or lymphocyte activation gene 3 (anti-LAG-3), have demonstrated substantial and durable clinical benefit. For example, anti-PD-1 monotherapy has shown a median overall survival of 36.9 months and a 5-year overall survival (OS) of over 40% [1,2]. In particular, the combination of nivolumab and ipilimumab has achieved a median OS of 71.9 months and a 10-year OS of 43% [3] according to the last follow-up results from the Checkmate 067 study [3]. Recently, the first-line anti-PD-1 and anti-LAG-3 combination, nivolumab and relatlimab, also reported encouraging results, showing improved median progression-free survival (PFS), OS, and overall response rate (ORR) with the combination compared to nivolumab alone [4].

Despite these advances, the optimal duration of ICI therapy remains undefined and is not clearly addressed in current guidelines [5,6,7]. Prolonged treatment raises concerns regarding immune-related adverse events (irAEs), diminished quality of life, and healthcare burden. Biomarker-driven approaches may help guide individualized therapy durations without compromising long-term outcomes [8].

Circulating tumor DNA (ctDNA) has emerged as a promising, minimally invasive biomarker in melanoma. Detection rates at baseline are high, up to 80% using digital droplet PCR (ddPCR) [9,10], and ctDNA levels correlate with tumor burden and clinical response [11,12]. In particular, ctDNA clearance or sustained negativity during treatment has been associated with improved PFS and OS, while persistent positivity predicts progression and poorer overall outcomes [11,12,13,14,15,16,17]. Although early approaches focused on hotspot mutations (e.g., BRAFV600E) detected by ddPCR, more comprehensive next-generation sequencing (NGS) platforms have expanded mutation coverage and enabled longitudinal tumor tracking [17,18,19,20,21,22]. Tumor-informed assays, based on whole-exome or whole-genome sequencing of individual tumors, offer high specificity and sensitivity for molecular residual disease (MRD) detection [23,24].

Recent studies suggest that personalized, tumor-informed ctDNA assays can stratify patients based on treatment response and prognosis [25,26]. However, evidence in advanced melanoma remains limited, and whether ctDNA can guide decisions on treatment duration is unclear. In this study, we evaluated whether early ctDNA clearance or persistent negativity can identify patients with advanced melanoma likely to derive long-term benefit (≥2 years) from anti–PD-1-based therapy.

## 2. Materials and Methods

### 2.1. Subjects and Study Design

This was an exploratory, single-center study including patients with stage IV melanoma treated with anti–PD-1-based therapy (monotherapy or in combination with anti–CTLA-4 or other agents) prospectively enrolled between January 2021 and October 2022. ctDNA data was analyzed retrospectively and patients were included in the analysis if they were treated with anti-PD-1 therapy, either as monotherapy or in combination with other agents, and if they had at least one on-treatment ctDNA test result. Longitudinal blood samples were collected prior to or at the time of ICI initiation and every 2–4 months thereafter for clinical ctDNA testing, for up to 29 months. Imaging (CT, and MRI where indicated) was performed per institutional practice. Subjects were identified in clinic and clinical data were abstracted from medical records. The study was approved by the institutional ethics board and conducted in accordance with the Declaration of Helsinki.

Baseline clinical data were collected such as age, sex, ECOG performance status, staging according to American Joint Committee on Cancer 8th Edition (AJCC 8th), number of disease sites, lactate dehydrogenase (LDH) levels, tumor mutational status, type of anti-PD-1-based treatment initiated, and previous/subsequent therapies. Response to therapy was obtained from radiological imaging based on CT/MRI according to Response Evaluation Criteria in Solid Tumors version 1.1 (RECISTv1.1) criteria. The response was coded as complete response (CR), partial response (PR), stable disease (SD), and progressive disease (PD).

### 2.2. Biospecimen Collection

Formalin-fixed, paraffin-embedded (FFPE) tumor specimens were obtained from Hospital Clínic de Barcelona—August Pi i Sunyer Biomedical Research Institute Biobank (HCB-IDIBAPS Biobank) at any time before ICI therapy initiation, corresponding to biopsies of metastatic melanoma lesions (preferred) or primary melanomas. Samples were eligible if they had a minimum surface area of 5 × 5 mm^2^, a total tissue thickness of ≥60 μm, and tumor content ≥20%. Sections were mounted on glass slides and stained with hematoxylin and eosin for pathology review. Eligible samples were shipped to Natera, Inc. (Austin, TX, USA).

### 2.3. Personalized ctDNA Assay Using Multiplex PCR-Based NGS Workflow

Briefly, whole-exome sequencing (WES) was performed on tumor tissue and matched peripheral blood mononuclear cells (PBMCs) to identify patient-specific somatic single-nucleotide variants (SNVs). ctDNA detection and quantification were performed using a personalized, tumor-informed, 16-plex mPCR-NGS assay (Signatera^TM^, Natera, Inc., Austin, TX, USA), as previously described [27]. Longitudinal plasma samples were collected using 10 mL Streck tubes at baseline (pre-ICI or at treatment initiation) and at regular intervals during treatment and follow-up. While the collection of blood samples was performed prospectively and in real time, this was an exploratory study, and as such, for the ctDNA analyses were conducted retrospectively, after completion of clinical follow-up. Consequently, ctDNA data were not used for clinical decision-making within this cohort. A sample was classified as ctDNA-positive when ≥2 SNVs were detected. ctDNA levels were quantified and reported as mean tumor molecules per milliliter of plasma (MTM/mL), as MTM/mL remains more representative of true tumor burden, particularly in contexts of elevated cfDNA [19].

### 2.4. Statistical Analysis

The ctDNA statistical analysis plan was developed before the reconciliation of the laboratory and de-identified clinical data. Descriptive statistics were used to summarize the clinical characteristics of cases, with median and range for continuous variables and frequency and percentage for categorical variables. The primary outcome measure was PFS, assessed between the date of ICI treatment initiation and the date of radiologic and/or clinical disease progression or death. The Kaplan–Meier method was used for estimating the survival distributions. Log-rank test or Cox proportional hazards model was used for comparing two survival distributions, with *p* ≤ 0.05 being considered significant. The OS analysis was restricted to patients receiving first-line therapy for metastatic disease. Statistical analyses were performed in STATA (v16.1) and R (version 4.3.1).

Landmark analyses were performed at 6 and 9 months post-treatment initiation and compared those that had a ctDNA-negative status or demonstrated clearance versus patients who were ctDNA-positive at each landmark. Patients who had a PFS event within the landmark window (6 months or 9 months) were excluded from the analyses accordingly. Landmark analyses evaluated associations between ctDNA dynamics and clinical outcomes (PFS and OS) at 6 and 9 months. Several patients contributed ctDNA samples at multiple timepoints (6 and 9 months), leading to overlap between the landmark analysis groups.

## 3. Results

A total of 56 patients with advanced melanoma were enrolled between January 2021 and October 2022. Personalized ctDNA assays were designed for 31 out of 56 patients (55.3%) who had sufficient tumor tissue and subsequent plasma timepoints available for analysis. Of these, three patients were excluded because they received targeted therapy. Across the cohort, 137 longitudinal plasma samples were analyzed.

### 3.1. Patient and Tumor Characteristics

**Figure 1:**
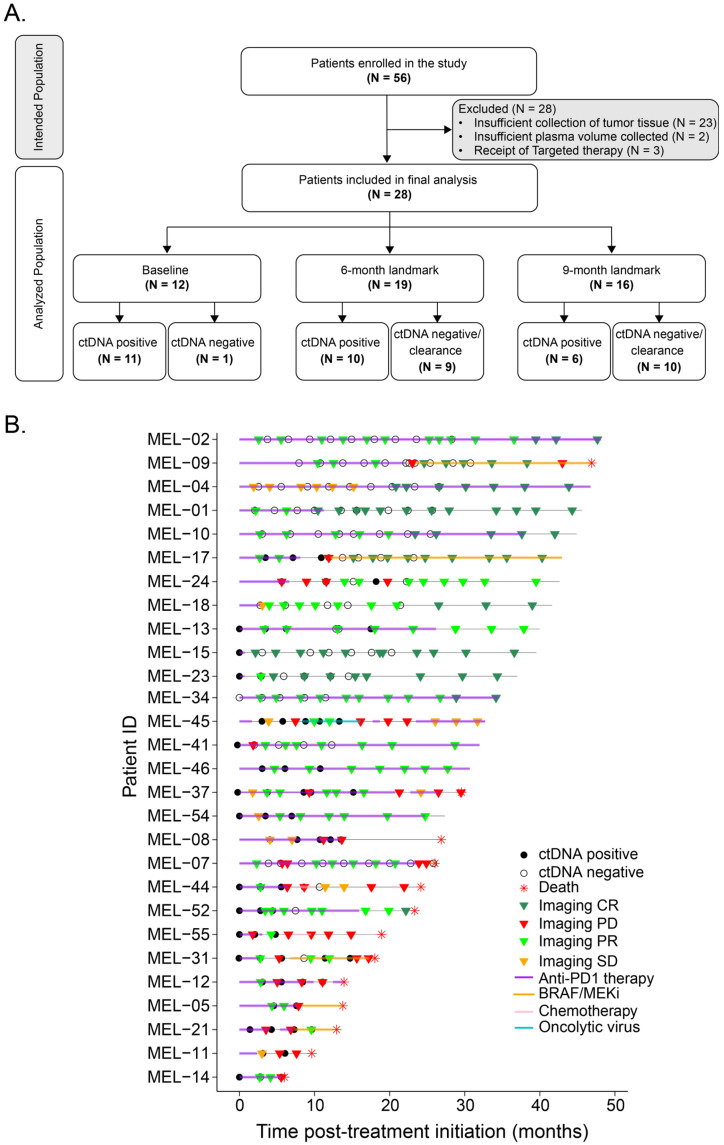
Flow diagram and overview plot of patients included in the study. (**A**) Flow diagram of patient inclusion criteria for analysis. (**B**) Overview plot showing ctDNA findings, imaging outcomes, and initial and subsequent treatment data from our cohort of advanced melanoma patients treated with anti-PD-1-based therapy. CR: Complete response; PD: Progressive disease; PR: Partial response; SD: Stable disease.

Baseline characteristics were found to be well-balanced between the enrolled (*N* = 56) and analyzed (*N* = 28) cohorts (Table 1). Among the 28 analyzed patients, 42.9% received anti-PD-1 + anti-CTLA-4, 25% received anti-PD-1 monotherapy, and 32.1% received anti-PD-1 in combination with other agents for advanced disease. Five patients had previously received anti–PD-1 therapy in the adjuvant setting. Of these, three initiated their current treatment for advanced disease within 3 months of completing adjuvant therapy, while the other two started 13 and 14 months later, respectively. Eight patients had received previous BRAF/MEK inhibitors (BRAF/MEKi), 7 in the metastatic setting, and 1 in the adjuvant setting, 6 of them within ≤3 months before starting current treatment (Table 1). Pre-ICI ctDNA test results were available for 12/28 patients (42.8%). Longitudinal on- and post-treatment plasma samples (*N* = 125) were collected from all 28 patients (Figure 1A). The complete clinical course for all patients is depicted in Figure 1B.

At the 24-month follow-up, 21 of 28 evaluable patients had discontinued therapy: 16 due to disease progression, 3 due to immune-related toxicity, 1 for non-melanoma causes, and 1 due to a planned discontinuation after 2 years. Despite this high discontinuation rate (72.4%), 12 patients remained progression-free. Median follow-up was 31 months (range, 21–40.2).

### 3.2. ctDNA Dynamics Are Predictive of Response to First-Line Anti-PD-1-Based Treatment

Pretreatment ctDNA-positivity rate was 91.7% (11/12) with ctDNA levels ranging from 0.58 to 82.07 MTM/mL (median 6.39 MTM/mL). Positivity rates during ICI were as follows: 36.4% (4/11) at 3 months; 64% (16/25) at 6 months, 52.6% (10/19) at 9 months; 43.75% (7/16) at 12 months; 30.8% (4/13) at 15 months, 18 months, and 24 months; and 27.3% (3/11) at 29 months.

**Figure 2:**
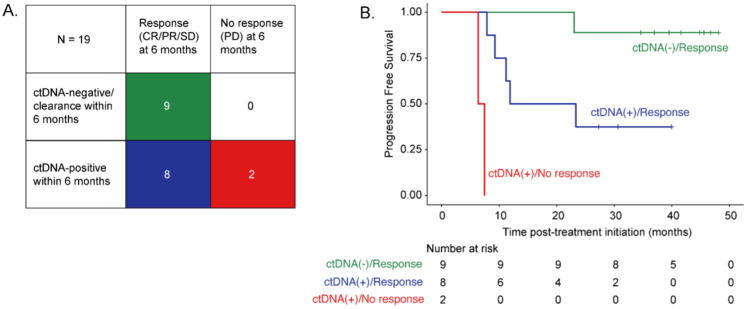
(**A**) Radiographic response status at 6 months (columns) and ctDNA status within 6 months (rows) are shown for 19 patients. (**B**) Kaplan-Meier estimates of progression-free survival among patients with at least one ctDNA measurement within 6 months from ICI initiation stratified according to 6-month radiographic response status and ctDNA status.

Among patients with pre-treatment ctDNA-positivity and available samples at 6 months (*N* = 7) and 9 months (*N* = 6), ctDNA clearance was observed in 33.3% (2/6) and 50% (3/6), respectively. Median time to ctDNA clearance at 6 months was 2.95 months (2.87–3.03 months) and 3.03 months (range 2.87–7.46 months) at 9 months. All patients with ctDNA-negativity/clearance at 6 months (*N* = 9) achieved radiographic response (CR/PR/SD) at the 6-month assessment and had a superior PFS compared to ctDNA-positive patients, irrespective of their radiographic response (Figure 2). Furthermore, 89% of patients with ctDNA negativity/clearance at the 6-month landmark showed sustained clinical benefit over 24 months.

### 3.3. Landmark Analysis at 6 Months and 9 Months: Association Between ctDNA Status and Long-Term Clinical Outcomes of Anti-PD-1-Based Therapy

**Figure 3:**
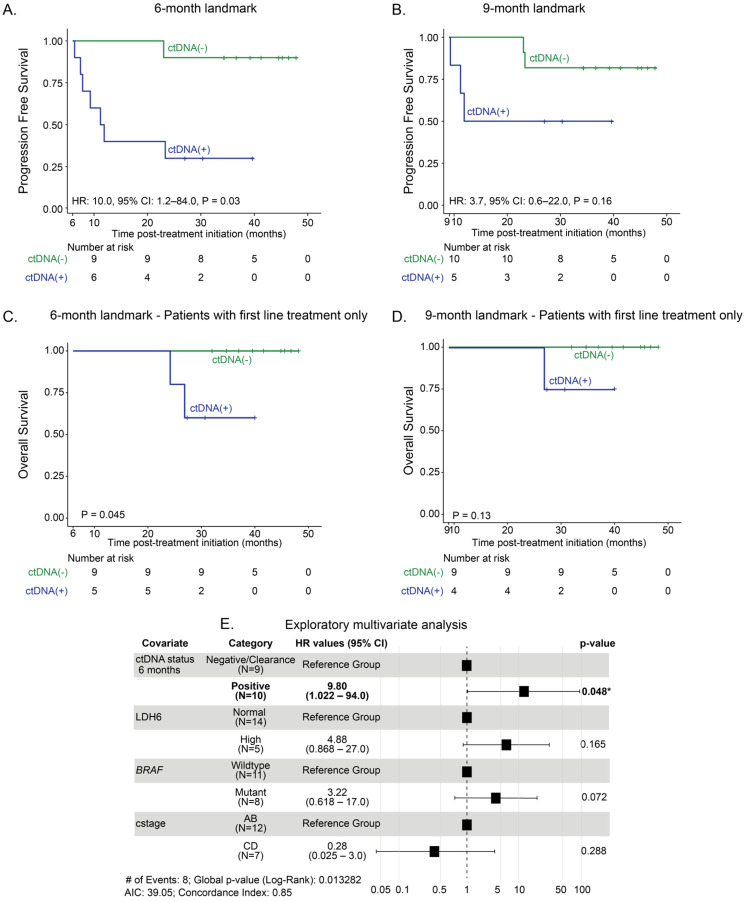
Clinical outcomes and ctDNA status at the landmark timepoints. Kaplan-Meier estimates of patients with advanced melanoma representing progression-free survival stratified by ctDNA status at the (**A**) 6-month landmark and (**B**) 9-month landmark. Kaplan-Meier estimates of overall survival stratified by ctDNA-status at the (**C**) 6-month landmark and the (**D**) 9-month landmark in patients receiving first-line treatment. (**E**) Multivariate Cox regression model for identifying predictors of PFS incorporating baseline covariates and ctDNA status at 6 months. The table displays each covariate, its hazard ratio (HR) 95% confidence interval (CI), and corresponding *p*-value. Abbreviations: AIC, Akaike Information Criterion; *BRAF*, *BRAF* mutational status; cstage, M stage according to AJCCv8; LDH6, LDH at 6 months within normal range or above. * represents statistical significance at *p* < 0.05.

Since we observed that ctDNA positivity in patients receiving anti-PD-1-based therapy reflects unfavorable patient outcome, we next examined the value of serial ctDNA sampling for progression and survival prognostication across 6 and 9 months. At the 6-month landmark, patients with ctDNA negativity had significantly improved PFS compared to those with ctDNA positivity (HR: 10.0, 95% CI: 1.2–84.0, *p* = 0.03; Figure 3A). The median PFS for the ctDNA-negative group was not reached, where it was 11.6 months for patients who were ctDNA-positive. When assessing OS in the 6-month group, ctDNA-positivity was associated with inferior OS compared with ctDNA-negativity (*p* = 0.045, Figure 3C). The median OS was 46.9 months for ctDNA-negative patients and not reached for the ctDNA-positive group.

At the 9-month landmark, no significant differences in PFS were observed between ctDNA-positive and ctDNA-negative groups (HR = 3.7, 95% CI 0.6–22.0, *p* = 0.16; Figure 3B). The median PFS was not reached in the ctDNA-negative group and 11.9 months in the ctDNA-positive group. Similarly, OS did not differ significantly between groups (*p* = 0.13; Figure 3D).

A multivariate model incorporating LDH levels, BRAF status, clinical stage, and ctDNA status at 6 months identified ctDNA positivity as an independent predictor of shorter PFS (Figure 3E). These findings support the additive prognostic value of molecular markers for early and long-term anti-PD-1-based therapy outcome stratification.

### 3.4. Clinical Case Examples

#### 3.4.1. Patient 1: Case MEL-17

**Figure 4:**
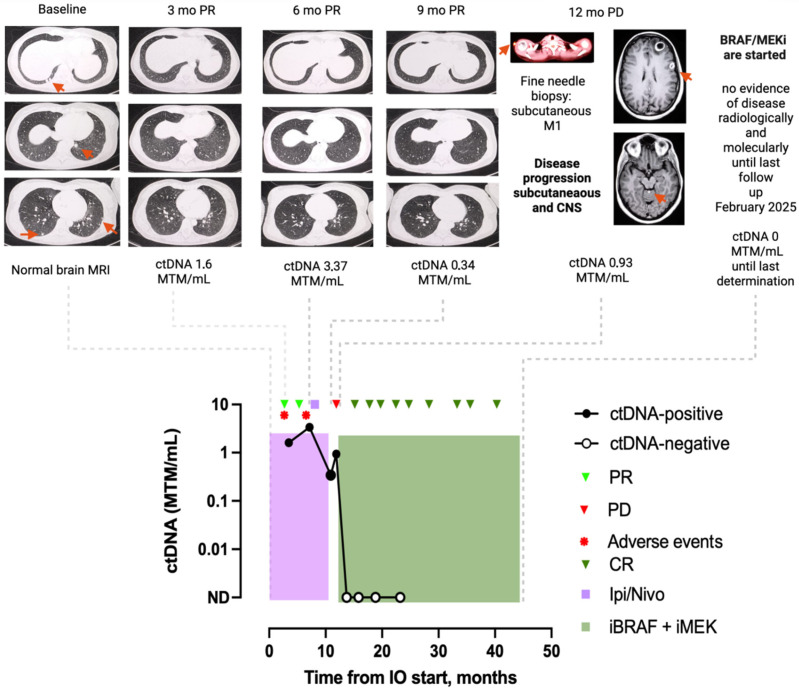
Graphic representation of clinical case MEL-17: Persistent ctDNA positivity preceding PD on anti-PD-1 therapy; ctDNA clearance upon switching to targeted therapy concordant with CR. This patient was initially diagnosed with oligometastatic lung disease from advanced BRAF-mutant melanoma, without CNS disease (stage IVb), and underwent first-line treatment with ipilimumab and nivolumab combination. ctDNA was persistently positive on anti-PD-1 therapy; ctDNA increase preceded progression with new subcutaneous and intracranial disease. She was subsequently started on second-line BRAF/MEKi, and ctDNA cleared after switching to targeted therapy with CR. All ctDNA-positive assessments are represented by filled black circles, and ctDNA-negative assessments by open black circles. Red arrow = metastatic lesion location, red circle = adverse events, red triangle = progression disease, pale green triangle = partial response, dark green triangle = complete response, purple square = ipilimumab and nivolumab combination treatment, green square = BRAF and MEK inhibitors combination treatment. Abbreviations: BRAF/MEKi, BRAF and MEK inhibitors; CNS, central nervous system; CR, complete response; PD, progressive disease; PR, partial response; Ipi/Nivo, ipilimumab and nivolumab combination treatment; IO, Immunotherapy. Created in BioRender. Martinez-Vila, C. (2025) https://BioRender.com/5xuybc5.

A 35-year-old woman with stage IVb BRAFV600-mutant melanoma and multiple lung metastases at initial diagnosis received first-line treatment with a combination of nivolumab 1 mg/kg and ipilimumab 3 mg/kg (Figure 4). During the first 9 months of therapy, the patient had a sustained PR on imaging despite persistently positive ctDNA results (1.6 MTM/mL at 3 months, 3.37 MTM/mL at 6 months, and 0.34 MTM/mL at 9 months). A pre-ICI treatment ctDNA test result was not available. Treatment was temporarily discontinued after 6 months due to grade 4 gastrointestinal toxicity. At 12 months post-initiation of ICI treatment, she presented with a new palpable lesion, confirmed by incisional biopsy to be a subcutaneous metastasis. A restaging brain MRI also revealed three new intracranial metastases. The extracranial and intracranial PD was concurrent with a small increase in ctDNA (from 0.34 to 0.93 MTM/mL). The patient subsequently initiated BRAF/MEKi therapy, resulting in ctDNA clearance within 3 months and a durable (42 months) intracranial and extracranial CR.

#### 3.4.2. Patient 2: Case MEL-15

**Figure 5:**
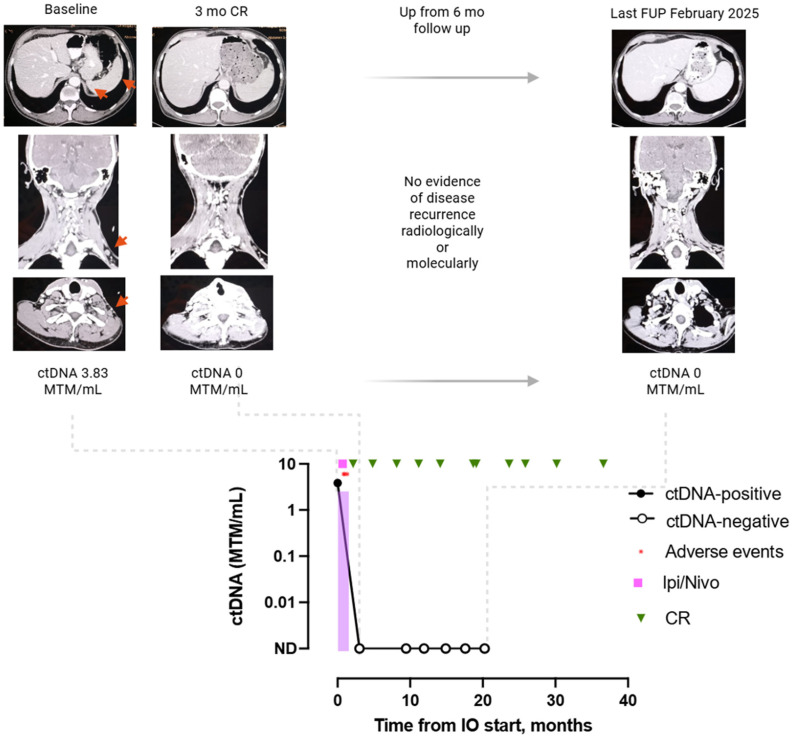
Graphic representation of clinical case MEL-15: Sustained ctDNA clearance post anti-PD-1 therapy was concordant with CR during surveillance/post-treatment monitoring. This patient was diagnosed with advanced RAS-mutant melanoma with small-volume disease in the splenic and subcutaneous sites (IVc). ctDNA was available and positive prior to treatment initiation. He was treated with ipilimumab and nivolumab with a severe hepatic irAE with early treatment discontinuation. Since the first longitudinal on-treatment assessment, he presented with a ctDNA clearance that persisted until the last assessment. According to radiological imaging, he maintained a CR until the last follow-up. All ctDNA-positive assessments are represented by solid black circles, and ctDNA negative assessments by open black circles. Red arrow = metastatic lesion location, red circle = adverse events, pink square = ipilimumab and nivolumab combination treatment, green triangle = CR. Abbreviations: CR, complete response; FUP, follow-up; Ipi/Nivo, ipilimumab and nivolumab combination treatment; IO, Immunotherapy. Created in BioRender. Martinez-Vila, C. (2025) https://BioRender.com/8gfkzqf.

A 64-year-old man with stage IVc NRAS-mutant melanoma presented with splenic and left supraclavicular lymph node metastases (Figure 5). The patient received combination therapy with nivolumab 1 mg/kg and ipilimumab 3 mg/kg, but treatment was discontinued after the second cycle due to grade 4 irAE hepatotoxicity. Active surveillance was initiated. At the first radiologic assessment, 3 months after treatment initiation, he achieved a CR, which was maintained through the most recent follow-up. Concurrently, ctDNA was negative from the first on-treatment measurement (3.83 MTM/mL at the pre-treatment timepoint to 0 MTM/mL at 3 months since treatment start) and has remained negative throughout follow-up.

## 4. Discussion

Despite the important advances in treatment strategy in advanced melanoma, 35% of patients experience primary resistance to treatment, 25–45% develop acquired resistance, and all are at risk for treatment-related toxicities [1,2,3,4,5,6]. Although clinical prognostic factors such as LDH levels remain relevant, there are no validated biomarkers that reliably predict which patients will derive durable benefit from ICIs. Identifying such biomarkers would allow for individualized treatment duration and safer discontinuation strategies [8].

ctDNA has emerged as a minimally invasive and dynamic biomarker capable of tracking tumor burden in real time [10,13,14,15,16,17,18,19,20,21,22,23,24,25,26,27]. Prior studies have shown that ctDNA levels correlate with radiologic assessments such as CT and PET/CT, and that increases in ctDNA are associated with shorter OS and PFS [10,13,14,15,16,17,18,19,20,21,22,23,24,25,26,27,28]. Notably, ctDNA has demonstrated superior prognostic performance compared to LDH in multiple studies [29,30].

In this study, a total of 56 patients were prospectively enrolled, and 28 patients (50%) were analyzed, which highlights the practical challenges of biomarker research in a high-volume academic and clinical center like ours. However, all participants were identified prospectively. Baseline characteristics were well balanced between patients with analyzable ctDNA and the overall enrolled cohort, supporting the representativeness of the analyzed population. Our study utilized a personalized, tumor-informed ctDNA assay to assess the prognostic value of ctDNA status and dynamics in patients receiving anti–PD-1-based therapy. This 16-plex mPCR-NGS assay is based on whole-exome sequencing of tumor tissue and paired germline DNA, enabling the selection of patient- and tumor-specific SNVs. Additionally, this approach filters out clonal hematopoiesis and enhances sensitivity through deep sequencing. Using this approach, we observed a high ctDNA detection rate at baseline (*N* = 11/12, 91.7%), which is substantially higher when compared to previously reported studies using ddPCR and other NGS assays [9,10,11,12].

We observed that ctDNA positivity at 6 months was strongly associated with inferior PFS and OS. In contrast, 89% (8/9) patients with ctDNA negativity at this timepoint remained progression-free for at least 2 years. The 6-month ctDNA status emerged as the most robust independent predictor of long-term benefit in multivariate analysis. Although ctDNA dynamics at 9 months were not significantly associated with PFS or OS, a smaller sample size may have limited statistical power. Importantly, we found that rising ctDNA levels within first 6 months were significantly associated with a lack of clinical response at 6 months to anti-PD-1-based therapy. These findings mirror earlier reports showing that ctDNA clearance after only a few cycles of ICI predicts clinical benefit [16]. Other studies using the same tumor-informed assay have shown similar results across solid tumors and treatment types [16,17,27,31], including melanoma [25,26]. For example, early ctDNA clearance has been linked to improved PFS and OS, and ctDNA dynamics appear to be important in predicting response (reviewed in [14]).

Limitations to this study include the small sample size, incomplete pre-ICI treatment ctDNA sampling, and a median follow-up of 31 months, which may still be too short to fully capture outcomes in long-term responders. Nonetheless, our findings support the use of personalized ctDNA monitoring as a complementary biomarker to imaging for early identification of long-term responders. Importantly, we propose that ctDNA clearance or persistent negativity at 6 months may serve as a surrogate endpoint for durable clinical benefit. These results warrant prospective validation in larger cohorts. Future studies should assess whether ctDNA-guided treatment discontinuation strategies can reduce exposure to immunotherapy without compromising outcomes, as well as whether ctDNA-guided treatment changes improve clinical outcomes in patients failing to clear ctDNA.

## 5. Conclusions

Dynamic, personalized ctDNA monitoring may help identify patients who can safely discontinue anti–PD-1 therapy after achieving molecular response. ctDNA negativity at 6 months was associated with excellent 2-year outcomes, suggesting its potential as a surrogate biomarker of long-term benefit. While further validation and evaluation of earlier on-treatment timepoints are needed, this study provides a framework for incorporating molecular monitoring into future treatment de-escalation and personalization trials.

## Figures and Tables

**Table 1:** Clinical Characteristics of the analyzed (*N* = 28) and intended (*N* = 56) patients prior to ICI initiation.

Characteristic, *N* (%)	Analyzed, *N* = 28	Enrolled, *N* = 56	*p* Value ^1^
Mean age, years (range)	63.61 (29–98)	66.54 (29–98)	0.085
Sex, male	12 (42.9)	27 (48.2)	0.926
ECOG ^2^			0.087
0	18 (64.3)	35 (62.5)	
1	10 (35.7)	18 (32.1)	
2	0 (0)	3 (5.4)	
M stage (AJCCv8)			0.0612
IVA	11 (39.3)	16 (28.6)	
IVB	6 (21.4)	9 (16.1)	
IVC	4 (14.3)	16 (28.8)	
IVD	7 (25)	15 (26.8)	
Number of disease sites			0.289
<3	23 (82.1)	40 (71.4)	
≥3	5 (17.9)	16 (28.6)	
LDH levels			0.233
≤ULN ^3^	21 (75)	40 (71.4)	
>ULN	7 (25)	16 (28.6)	
Mutational status ^4^			
BRAF-non mutant	16 (57.1)	33 (58.9)	0.684
BRAFV600	12 (42.9)	22 (39.3)	0.677
NRAS mutant	5 (27.8)	12 (21.4)	0.397
Efficacy ^5^			**0.0177**
SD	3 (10.7)	6 (10.7)	
PR	15 (53.5)	29 (51.8)	
CR	7 (25)	11 (19.6)	
PD	3 (10.7)	10 (17.9)	
Current treatment			0.575
AntiPD1 + antiCTLA4	12 (42.9)	18 (32.1)	
AntiPD1 + other	7 (25)	14 (25)	
AntiPD1 alone	9 (32.1)	21 (37.5)	
BRAF/MEKi	0 (0)	3 (5.4)	
Previous anti-PD1-based therapy			0.454
Adjuvant setting	5 (100)	9 (75)	
Metastatic setting	0 (0)	3 (25)	
Previous BRAF/MEKi therapy			0.743
Adjuvant setting	1 (12.5)	1 (10)	
Metastatic setting	7 (87.5)	9 (90)	

^1^ Statistically significant *p* values are in bold. ^2^ ECOG = Eastern Cooperative Oncology Group. ^3^ ULN = upper limit of normal. ^4^ % calculated according to patients tested for mutational status. ^5^ CR= Complete response, PR = Partial response, SD = Stable disease, PD = Progressive disease.

## Data Availability

The authors declare that all relevant, non-proprietary data used to conduct the analyses are available within the article. To protect the privacy and confidentiality of patients in this study, clinical data are not made publicly available in a repository or the article but can be requested at any time from the corresponding author. All data shared will be de-identified.

## Abbreviations

The following abbreviations are used in this manuscript:

ctDNA	Circulating tumor DNA
MRD	Molecular residual disease
ICIs	Immune checkpoint inhibitors
PFS	Progression-free survival
OS	Overall survival
PD-1	programmed cell death-1
CTLA-4	Cytotoxic T-lymphocyte-associated protein 4
LAG-3	Lymphocyte activation gene 3
irAEs	Immune-related adverse events
CR	Complete response
PR	Partial response
PD	Progressive disease
SD	Stable disease
FFPE	Formalin-fixed paraffin-embedded
WES	Whole-exome sequencing
